# Medical History from the Earliest Times

**Published:** 1893-04-29

**Authors:** E. T. Withington


### Medical History from the Earliest Times.

By E. T. Withington, M.A., M.B. Oxon.

### LII.—MEDICAL PRACTICE IN THE SIXTEENTH CENTURY.

The noisy controversies, new systems, and important anatomical discoveries which mark the medical history of the Reformation age, tempt the historian to neglect what is after all his main object, the description of the actual practice of the period. But he has little excuse for so doing, for the "Hippocratic" physicians justified their title by composing many volumes of clinical histories and observations, which give us a vivid picture of the best aspect of our art at that period. Besides these, the "consilia," or letters of advice, still continued, though the writers are careful to assert that a physician is only justified in exceptional cases in prescribing for a patient he has never seen. As an interesting example of such a consilium, we may take the twenty-first "Medical Epistle" of the already-mentioned Dr. John Lange. "You complain to me, as to a faithful Achates, that your eldest daughter, Anna, is now marriageable, and has many eligible suitors, all of whom you are obliged to dismiss on account of her ill-health, the cause of which no doctor can discover: for one calls it cardialgia, a second palpitation, a third dyspnœa, a fourth hysteria, nor are there wanting who say that her liver is out of order. Wherefore you entreat me by our ancient friendship to give an opinion on her case, with advice as to marriage, and you send me an excellent account of her symptoms. Her face, which last year showed rosy cheeks and lips, has become pale and bloodless, her heart palpitates at every movement, and the pulse is visible in the temporal arteries; she loses her breath when dancing or going upstairs, she dislikes her food, especially meat, and her legs swell towards evening, particularly about the ankles. I marvel that your physicians have not diagnosed the case from such typical symptoms. It is the affection, which the women of Brabant call the 'white fever,' or love sickness, for lovers are always pale, but there is very rarely any fever." He then discusses the pathology of the disease with copious Greek quotations from the Hippocratic treatise, "De Morbis Virginum," points out that Hippocrates recommends marriage, and says that, with the addition of simple purgatives and emmenagogues, he never knew it to fail. "So be of good cheer, marry your daughter, and I shall be glad to come to the wedding."

Among the earliest writers of "Observations" was Antony Benivieni, whose work "On the Hidden Causes of Diseases and Cures," quoted in a preceding article, though it strictly belongs to the fifteenth century, may best be considered here. Of the two following extracts, the first is the earliest known description of senile gangrene, while the second is a case perhaps unique in medical history.

(1) "Those who suffer from the black ulcer, which the Greeks call gangrene, if it begins from the toe, and the patients are old or in broken health, die rapidly. I knew Cambinus, Charles, and Thomas, citizens of Florence, and very many others affected by this disease, and they all died in a short time. The flesh gradually turns dark or livid, and sometimes dries up. The adjacent skin loses its sensibility, becomes pale or livid, and covered with black swellings. The disease creeps on till it affects the bone, and if you amputate, even through healthy flesh it will return again in the stump. (2) A monk of the Order of St. Augustine at Florence came to me and complained that the bone of his head was wearing away daily. Surprised at this I felt his head, and found that the fore part was almost entirely boneless; and what was most amazing, there was no disease of the skin or flesh. Nothing did him any good, and in a few years he died, having lost nearly all his skull. Benivieni suggests that it may have been due to some fine acrid humour which, passing through the pores of the flesh, eroded the harder bone, just as a flash of lightning may melt the gold in a purse without injuring the latter.

One of the most famous physicians of the sixteenth century was Amatus Lusitanus, a Portuguese Jew, who, with some hundreds of his nation, had been forcibly baptised in childhood. He continued, however, to practise the religion of his ancestors in secret, and might perhaps have done so in security, had he not published a commentary on Dioscorides, pointing out the mistakes in the famous edition of that author, by Matthioli of Siena; and the enraged botanist, unable to refute his adversary, loudly accused him of apostasy. We have seen how Saladin's judges treated a similar charge in the case of Maimonides; but the Christian theologians thought differently, and they hunted the unfortunate Amatus from place to place like a wild animal, till he found refuge at last among the Turks at Salonica. In one of his many flights he lost all his manuscripts, but fortunately recovered the most important, the first five "Centuries" of his "Medical Cures," to which he afterwards added two hundred more. The following are among the more interesting of the 700 cases, and are given, for the sake of brevity, without comment.

"A girl whom I had cured of aphthæ, or ulcers of the tongue, fell down stairs, and her skull over the right temple was bent inwards, leaving a large depression, but with no apparent wound or fracture. I sent for a surgeon, and ordered him to apply a cupping glass over the spot, and made a plaster of white of egg and astringent powders on lint, and in a few days she was well. I suppose all know that the bones of children are thinner and more full of blood than those of adults (see Hip. 'On Head Injuries'), and therefore are easily bent but hardly broken; as appeared also in the son of a tanner, who lived with Malamtesta the colourman, who fell from a height and bent in his skull but was cured in the same way." "A boy swallowed a brass coin, and discharged it a year afterwards, so worn away by the digestive power that all marvelled, for the innate heat is much greater in the young. I knew a man at Ferrara who ate leather, shells, and broken glass, so that they called him the ostrich; for nature has given that bird the power of digesting everything, as I saw at Antwerp." "Melancholia—The daughter of Vincentio the tanner, aged 30, used, to wash her head and expose it in the hot sun in order to turn the hair red. By so doing she fell into an arthritis, or general pain of the joints, which we treated by bleeding from both arms, applying oil of roses and chamomile to the joints, and purgative draughts. But two months later, in the autumn, she washed herself again, put her head in the sun, and went mad. I ordered her to be bled from the cephalic vein to six ounces, cupping glasses to be applied to her shoulders, and friction to her arms and legs. Then, after large doses of senna, Indian electuary, and other cathartics to purge melancholia, she recovered; for when I ordered her hair to be cut off, she said she would rather die than suffer it; so we sent her to sleep by putting lettuce and poppy seed in her food." Amatus then distinguishes melancholia and mania, which are due, he says, to excessive heat of the black and yellow bile respectively. "Melancholia is first described by Homer, when he pictures Bellerophon wandering lonely on the Aleian plain, 'eating his heart and avoiding the paths of men'; but this was not a bad case, for we easily made her laugh." "A noble Frenchman, procurator of King Henry II. at Ragusa, got an affection, not of his reason, but of his imagination, for he fancied he had an abscess in his side, and offered great rewards to whoever would cure him. I found him in excellent health, with a rosy face and good pulse. He ate like a bull, drank like a Frenchman, and slept profoundly, but could in no wise be cured of his delusion, and proceeded to make his will and to give all he had to strangers, except one flagon, which he hugged, 'non secus ac elegantem puellam,' for it was full of generous wine." Amatus administered large doses of hellebore and senna to no purpose, till, remembering how Alexander of Tralles cured a patient who thought he had a serpent inside him, he determined to do likewise, and performed in imaginary operation with complete success on March 8th, 1557, the pus being represented by a mixture of hen's blood and milk. "The Turks treat gout by bringing in a goat to the patient and milking it over the painful limb, a remedy which, if effectual, should be borne in mind by all who seek wealth and honour." But diet is the great thing. "At Rome I treated Camillus, of the princely family of Colonna, for gout. On taking charge of the case I gave him full directions as to diet, which he swore to follow. But, *Deus bone*! hardly had he taken his sixth dose of purgative medicine than he ate a supper of fried eggs and all sorts of shell fish, not without punishment, for he was grievously tormented all night. As soon as I dismounted from my mule next morning his servants ran up and told me all about it. (Always find out as much as you can from a patient's servants.) So, when he complained, I accused him of having broken his promise, &c." In short, doctor and patient had a regular quarrel. "And I would not have gone back to him again; no, not if he had offered me one of his estates."

